# Management Based on Multimodal Brain Monitoring May Improve Functional Connectivity and Post-operative Neurocognition in Elderly Patients Undergoing Spinal Surgery

**DOI:** 10.3389/fnagi.2021.705287

**Published:** 2021-07-15

**Authors:** Shuyi Yang, Wei Xiao, Hao Wu, Yang Liu, Shuai Feng, Jie Lu, Tianlong Wang

**Affiliations:** ^1^Department of Anesthesiology, Xuanwu Hospital, Capital Medical University, National Clinical Research Center for Geriatric Disease, Beijing, China; ^2^Department of Neurosurgery, Xuanwu Hospital, Capital Medical University, Beijing, China; ^3^Department of Radiology, Xuanwu Hospital, Capital Medical University, Beijing, China

**Keywords:** management based on multimodal brain monitoring, elderly patients, spinal surgery, perioperative neurocognitive disorders, functional connectivity, systemic inflammatory response

## Abstract

Perioperative neurocognitive disorder (PND) is a common condition in elderly patients undergoing surgery. Sedation, analgesia, regional cerebral oxygen saturation (rSO_2_), and body temperature are known to be associated with PND, but few studies have examined the contribution of these factors combined in detail. This prospective, randomized, controlled, double-blinded study investigated whether anesthesia management based on multimodal brain monitoring—an anesthesia management algorithm designed by our group—could improve the post-operative cognitive function and brain functional connectivity (FC) in elderly patients undergoing elective spinal surgery with general anesthesia. The patients (aged ≥65 years) were randomized into two groups [control (Group C), *n* = 12 and intervention (Group I), *n* = 14]. Patients in Group I were managed with multimodal brain monitoring (patient state index, spectral edge frequency, analgesia nociception index, rSO_2_, and temperature), and those in Group C were managed with routine anesthesia management. All patients were pre- and post-operatively evaluated (7 days after surgery) with the Montreal Cognitive Assessment (MoCA). Amplitude of low-frequency fluctuation (ALFF) and FC were analyzed after resting-state functional MRI. Serum C-reactive protein (CRP) and lipopolysaccharide levels were measured, and the correlation between FC and changes in inflammatory marker levels was analyzed. Mean post-operative MoCA score was higher in Group I (24.80 ± 2.09) than in Group C (22.56 ± 2.24) (*p* = 0.04), with no difference in PND incidence between groups (28.57 vs. 16.67%; *p* = 0.47). Group I also showed significantly increased ALFF values in several brain regions after surgery (*p* < 0.05), and FC between the left hippocampus and left orbital inferior frontal gyrus (FG), left middle FG, left superior temporal gyrus, and left precentral gyrus was enhanced (*p* < 0.05), which was negatively correlated with the change in serum CRP (pre vs. post-intervention) (*R* = −0.58, *p* = 0.01). These results suggest that management of elderly patients undergoing surgery by multimodal brain monitoring may improve post-operative neurocognition and FC by reducing systemic inflammation.

**Clinical Trial Registration:** http://www.chictr.org.cn/index.aspx, identifier: ChiCTR1900028024.

## Introduction

Perioperative neurocognitive disorder (PND), which includes neurocognitive complications, such as delirium and longer-lasting post-operative cognitive dysfunction, is common in elderly patients undergoing surgery under anesthesia (Subramaniyan and Terrando, 2019). The main symptoms of PND are memory and attention loss and reduced abilities in language comprehension and social adaptability (Le et al., 2014; Evered et al., 2018). In a recent study, the incidence rate of PND in elderly patients, who had undergone spinal surgery, was 43% (Zhang et al., 2018). Patients with PND have an increased risk of mortality and morbidity, longer hospital stays, significantly higher healthcare costs, and increased use of family care services (Monk et al., 2008; Steinmetz et al., 2009). With the aging of the global population, PND in elderly patients has become a major clinical problem.

Resting-state functional MRI (rs-fMRI) is a non-invasive method for examining the functional brain networks (Biswal et al., 1995). It analyzes the brain function and activity in the resting state (i.e., awake without thinking of anything in particular) based on the blood-oxygen level-dependent (BOLD) signal. rs-fMRI can distinguish patients with mild cognitive impairment (MCI) from healthy subjects (Chen et al., 2011) and Alzheimer's disease (AD) from normal aging (Khazaee et al., 2015). However, most rs-fMRI studies have focused on neurodegenerative diseases, such as AD and multiple sclerosis (Eijlers et al., 2019), and it has rarely been applied to the patients undergoing surgery, though it was recently demonstrated that the functional connectivity (FC) in certain brain areas may predict post-operative pain relief (Sawada et al., 2020). FC changes in PND—especially in elderly patients—have not been reported.

The PND is thought to arise from the neuroinflammation caused by stress from anesthesia and surgery (Lim et al., 2013). Biomarkers of the inflammation and stress [e.g., C-reactive protein (CRP) and cortisol] were found to be negatively correlated with the neuropsychological test score (Edipoglu and Celik, 2019). Inadequate sedation during the surgery is related to an increased stress response, which can cause the release of inflammatory cytokines and lead to the cognitive dysfunction (Vlachakis et al., 2017; Quan et al., 2019). Similarly, the oversedation, acute pain, cerebral hypoxia, and hypothermia can exacerbate neuroinflammation and are correlated with post-operative cognitive deficits (Drabek et al., 2014; Gong et al., 2018; Koyama et al., 2019; Snyder et al., 2021), which can potentially be prevented by monitoring the sedation [e.g., by electroencephalography (EEG)], analgesia, and regional cerebral oxygen saturation (rSO_2_) (Murniece et al., 2019; Bocskai et al., 2020; Cotae et al., 2021). Accordingly, we developed a multimodal brain monitoring management strategy involving EEG-based patient state index (PSI) and spectral edge frequency (SEF) combined with other monitoring tools, such as the analgesia nociception index (ANI), rSO_2_, and nasopharyngeal temperature. The anesthesia management strategy was based on the assumption that maintaining the monitoring parameter values in a normal range would alleviate the cognitive dysfunction. However, whether it is more effective in preventing PND than routine anesthesia management—which depends solely on the experience of an anesthesiologist and does not include monitoring of PSI, SEF, ANI, and rSO_2_–is unknown.

In this study, we investigated the effect of anesthesia management by multimodal brain monitoring on post-operative cognitive function in elderly patients undergoing spinal surgery with general anesthesia. We also examined the correlation between inflammatory markers and FC in cognition-related brain regions in order to clarify the possible mechanism underlying post-operative changes in cognition. We hypothesized that patients receiving this intervention would have higher neuropsychological test scores, improved FC in the brain, and lower serum levels of inflammatory biomarkers compared to those who underwent routine anesthesia management.

## Methods

### Study Design

This prospective, randomized controlled trial with patient and outcome assessor blinding was approved by the institutional review board of Xuanwu Hospital, Capital Medical University and registered with the Chinese Clinical Trial Registry (ChiCTR1900028024). The trial was conducted from September 2019 to December 2020. All experimental procedures were carried out in accordance with the Declaration of Helsinki. The verbal and written informed consents were obtained from all participants.

### Patients

The inclusion criteria for patients were as follows: age ≥65 years, an American Society of Anesthesiologists (ASA) physical status classification of I or II, scheduled for elective spinal surgery, and at least 6 years of education. Exclusion criteria were as follows: participation in other trials, diseases associated with cognitive impairment, preoperative Montreal Cognitive Assessment (MoCA) score <19, severe mental disorders, body mass index (BMI) >35 kg/m^2^, contraindication for study medications or MRI, history of neurosurgery or head trauma, persistent arrhythmia, alcohol or drug use, left-handedness, and refusal to participate.

### Randomization and Blinding

Patients were randomized into intervention and control groups (Groups I and C, respectively) using a random digit table generated using an online software program (https://tools.medsci.cn/rand) by an investigator blinded to this study. A complete concealment method was used for random assignment: grouping information from the random digit table was prepackaged in consecutively numbered, sealed, opaque envelopes. When a patient who met the inclusion criteria was enrolled, the anesthesiologist opened the corresponding envelope, and the patient received the treatment corresponding to their group. Patients and the outcome assessor were blinded to group assignment. Except in cases of acute complications, the randomization code was discarded only after patient enrollment and follow-up had ended.

### Anesthesia Management

Heart rate, blood pressure, pulse O_2_ saturation level, nasopharyngeal temperature, end-tidal CO_2_ level, urine output, and other parameters were routinely monitored. A standard general anesthesia induction protocol (0.15 mg/kg etomidate, 0.8 mg/kg rocuronium, and 0.3 μg/kg sufentanil) was used in all patients, who were then intubated with an endotracheal tube and ventilated with a 50% O_2_-air mixture. The initial dosages of maintenance anesthesia were 4 mg/kg/h propofol and 0.2 μg/kg/min remifentanil. The intervention was implemented during anesthesia maintenance according to the group. The initial dosage of norepinephrine was set as 0.03 μg/kg/min, and the infusion rate was increased or decreased in increments of 0.02 μg/kg/min if the mean arterial pressure was <70 mmHg or raised >20% from the baseline value. Patients underwent intravenous patient-controlled analgesia (PCA) after surgery (40 mg oxycodone diluted to 100 ml with normal saline). PCA parameters were set as a bolus dose of 1 mg oxycodone with a lockout time of 5 min; the maximum amount within 1 h was 3 mg.

#### Management Based on Multimodal Brain Monitoring in Group I

**Figure 1:**
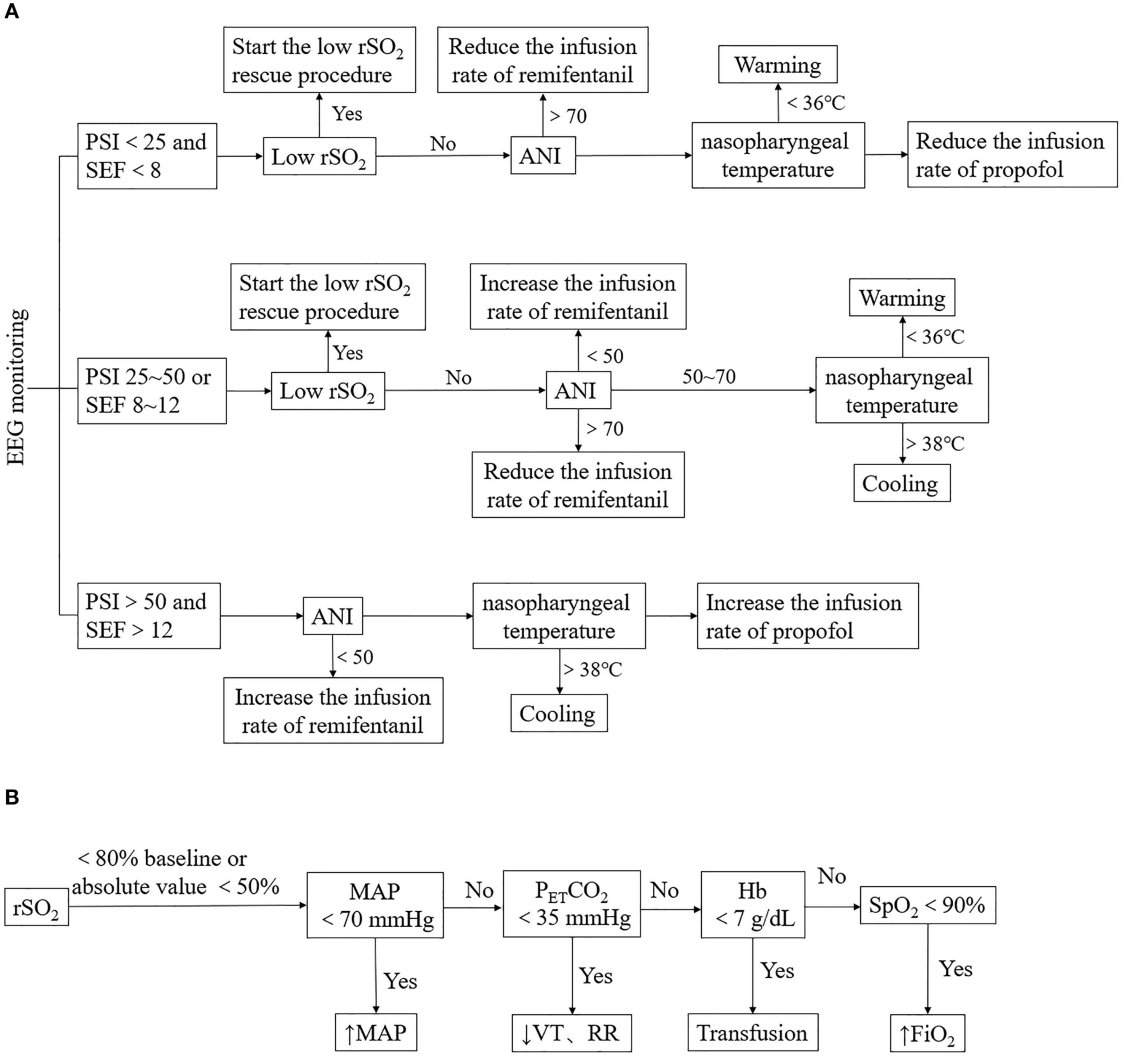
Anesthesia management of Group I. **(A)** Management based on multimodal brain monitoring. The management is triggered by PSI with the SEF, rSO_2_, ANI, and nasopharyngeal temperature were observed in sequence. Low rSO_2_ rescue procedure, adjustment of the infusion rate of remifentanil or propofol, warming or cooling was conducted according to the change of parameters. **(B)** Low rSO_2_ rescue procedure. MAP, P_ET_CO_2_, Hb, and SpO_2_ were adjusted when low rSO_2_ occurred. PSI, patient status index; SEF, spectral edge frequency; ANI, analgesia nociception index; rSO_2_, regional cerebral oxygen saturation; MAP, mean arterial pressure; P_ET_CO_2_, end-tidal carbon dioxide partial pressure; VT, tidal volume; RR, respiration frequency; Hb, hemoglobin; SpO_2_, pulse oxygen saturation; FiO_2_, fraction of inspiration oxygen.

Patients in Group I were managed with multimodal brain monitoring, which included the monitoring and regulation of sedation depth parameters (PSI and SEF), analgesia parameter (ANI), rSO_2_, and nasopharyngeal temperature. PSI, SEF, ANI, and rSO_2_ data were provided by the Root Platform (Masimo, Irvine, CA, USA). PSI (25–50) and SEF (8–12) were selected as the triggers for the intervention. The management protocol was as follows (Figure 1). (1) When both PSI and SEF values were abnormal, we checked the rSO_2_ value; if this had decreased by >20% from the baseline value, a low rSO_2_ rescue procedure was initiated (Figure 1B). (2) ANI was monitored, and the target range was set as 50–70. The infusion rate of remifentanil was increased or decreased in increments of 0.02 μg/kg/min when ANI was <50 or >70, until the ANI reached the target range. (3) Patients with hypothermia or hyperthermia (body temperature <36°C and >38°C, respectively) were treated by warming or cooling (target body temperature 36–38°C). If rSO_2_, ANI, and nasopharyngeal temperature were within the appropriate ranges but PSI and SEF values were still abnormal (PSI >50 and SEF >12 or PSI <50 and SEF <8), then the infusion rate of propofol was increased or decreased in increments of 0.5 mg/kg/h until the target ranges of PSI and SEF were reached. Finally, if PSI or SEF values were in the normal range but rSO_2_ remained abnormal, an rSO_2_ rescue procedure was initiated. If body movement occurred, analgesia and sedation were adjusted simultaneously.

#### Routine Anesthesia Management in Group C

Patients in Group C were managed with a routine anesthesia management protocol, in which the remifentanil infusion rate during maintenance depended on the experience and hemodynamic parameters of an anesthesiologist. Propofol was adjusted according to the bispectral index (BIS), with the target range set as 40–60. ANI and rSO_2_ were not monitored.

### Blood Sample Collection

Venous blood samples were collected 1 day before surgery (baseline, T_0_) and 24 h after surgery (T_1_). The samples were centrifuged for 15 min at 1,000 rpm, and the supernatant was frozen at −80°C until use. CRP and lipopolysaccharide (LPS) levels in the serum were detected by ELISA using the commercial kits (CRP: R&D Systems, Minneapolis, MN, USA; LPS:CUSABIO, Wuhan, Hubei, China) according to the instructions of the manufacturers.

### Patient Data Collection

The demographic and clinical data of patients, including age, BMI, ASA physical status, education level, history of hypertension, diabetes, coronary artery disease (CAD), operated segment, and preoperative diagnosis were recorded 1 day before surgery. The patients were preoperatively trained on how to use the numeric rating scale (NRS). Pain intensity was assessed using the NRS at 24 h after the surgery (T_1_). The dosage of anesthetics and vasoactive drugs and surgery and anesthesia duration were recorded during the surgery. The amount of oxycodone consumed, time of the first post-operative exhaust, adverse effects (nausea and vomiting), post-operative complications (e.g., wound infection or fever), and length of hospital stay were recorded after the surgery.

### Neuropsychological Assessment

Patients were evaluated with the MoCA 1 day before surgery (T_0_) and 7 days after surgery (T_2_) by the same neuropsychologist. To calculate the incidence of PND, we referred to previous practice effect data of community volunteers collected by our research group (Zhang et al., 2018): the practice effect was found to be 1.92 ± 1.19, and *Z*-score was calculated according to the formula [MoCA score (T_2_) – MoCA score (T_0_) – practice effect (mean)]/practice effect (SD); a diagnosis of PND was made if the *Z*-score was ≥1.96.

### rs-fMRI Data Acquisition

Patients underwent fMRI scanning 1 day before (T_0_) and 7 days after (T_2_) the surgery before neuropsychological assessment. The MRI data were obtained using a 3.0-T MRI scanner (Verio; Siemens Medical Solutions, Erlangen, Germany) with a 12-channel head coil. Head motion and scanner noise were minimized using cushions and earplugs. Participants were instructed to keep their eyes closed and remain relaxed with their heads still, and avoid thinking of anything in particular during the scan. The scanning parameters were as follows: repetition time (TR) = 2,000 ms, echo time (TE) = 30 ms, field of view (FOV) = 240 × 240 mm^2^, flip angle (FA) = 90°, section thickness = 4 mm, acquisition matrix = 64 × 64, voxel size = 3.8 mm × 3.8 mm × 4.0 mm, and 33 slices covering the whole brain. The sequence lasted for 488 s. Additionally, T1-weighted images were obtained with a three-dimensional magnetization-prepared rapid-gradient echo sequence, for which the parameters were as follows: TR = 1,900 ms, TE = 2.19 ms, inversion time = 900 ms, FA = 9°, FOV = 256 × 256 mm^2^, voxel size = 1.0 mm × 1.0 mm × 1.0 mm, and 176 slices at a thickness of 1.0 mm.

### Image Data Preprocessing

The Python-based pipeline Configurable Pipeline for the Analysis of Connectomes (https://fcp-indi.github.com) was used to preprocess image data; this was accelerated and simplified by the Beijing Intelligent Brain Cloud platform (http://www.humanbrain.cn).

Structural preprocessing involved the following steps: image deobliquing; right-to-left, posterior-to-anterior, and inferior-to-superior (RPI) reorientation; skull-stripping; normalizing individual skull-stripped brains to Montreal Neurological Institute 152 stereotactic space (1 mm^3^ isotropic) using linear and non-linear registrations; segmenting the brain into gray matter, white matter, and cerebrospinal fluid (CSF); and constraining the tissue segmentation of individual subjects by tissue priors from standard space obtained from the FMRIB Software Library (FSL; https://fsl.fmrib.ox.ac.uk/fsl/fslwiki).

Functional preprocessing was performed as follows. The first 10 time points were removed, and slice time correction was performed. The image was deobliqued and subjected to RPI reorientation. Motion parameters were obtained by motion correction to the average image. Skull-stripping was performed, and global mean intensity was normalized to 10,000. Functional images were registered to anatomical space by linear transformation, white matter boundary-based transformation, and prior white matter tissue segmentation from FSL. Motion artifacts were removed using independent component analysis - based strategy for automatic removal of motion artifacts (ICAAROMA) with partial component regression. The following nuisance signals were regressed out: mean values of the white matter and CSF signals derived from the prior tissue segmentations were transformed from anatomical to functional space; motion parameters (6 head motion parameters, 6 head motion parameters from a prior time point, and 12 corresponding squared items); linear trends; and a global signal for a single set of strategies.

### Amplitude of Low - Frequency Fluctuations (ALFF) Analysis

The time series for each voxel was filtered at 0.01–0.1 Hz and then converted to the frequency domain by the fast Fourier transform; the square root and average square root (ALFF) were obtained from each frequency of the power spectrum and each voxel. ALFF images were transformed to the standard space by applying the previous anatomical-to-standard-space registration. ALFF images were registered through smoothing [full width at half maximum (FWHM) = 6 mm] and *Z*-score standardization.

### Functional Connectivity Analysis

The primary outcome of this study (FC) was processed by seed-based whole-brain FC analysis; the seed region was selected from the results by comparing ALFF images in Groups I and C. The Pearson correlation coefficient between time series within the voxel and averaged time series in the seed region was used to define FC for each voxel. FC images were registered to standard space, and smoothing (FWHM = 6 mm) and Fisher's *Z* transformation were applied.

### Statistical Analysis

The sample size was estimated as previously described (Klaassens et al., 2019). At least 14 samples were needed from each group to detect a significant change in the rs-fMRI analysis. Assuming a 10% dropout rate, we planned to recruit 30 patients.

Statistical analyses were performed using SPSS v19.0 for Windows (IBM Corp., Armonk, NY, USA). Data are presented as mean ± SD, median, and interquartile range, or frequency as appropriate. The distribution of continuous outcome variables was tested for normality by the visual inspection and with the Kolmogorov–Smirnov test. Data that were normally distributed (demographic, anesthetic, and surgical data; neuropsychological assessment scores; serum CRP and LPS levels; time of the first post-operative exhaust; and length of hospital stay) were analyzed with the two-sample *t*-test. Data conforming to a non-normal distribution (operated segment, NRS score, and amount of oxycodone consumed) were analyzed with the Mann–Whitney *U*-test. Categorical data (ASA classification, history of hypertension, diabetes, CAD, and incidence of adverse effects and complications) were analyzed with the chi-squared test. Effect size was calculated using Cohen's *d* statistic, Cohen's *d* value of non-parametric data was transformed from η^2^. A *p* < 0.05 was considered statistically significant.

The two-sample *t*-test was used to analyze differences in ALFF and FC in the whole brain based on voxel levels between Groups I and C using the REST v1.21 software on the MATLAB 2014a platform (MathWorks, Natick, MA, USA). Gaussian random field (GRF) correction for multiple comparisons was applied to all statistical maps based on a voxel *p* < 0.05 and cluster *p* < 0.05. The relationships between FC value in significantly altered brain regions and changes in inflammatory marker expression after vs. before surgery were analyzed with Pearson's correlation coefficient. Effect size was calculated using Cohen's *f* statistic. Effect size in Pearson's correlation was represented by the *R*-value.

## Results

### General Characteristics of the Study Population

**Figure 2:**
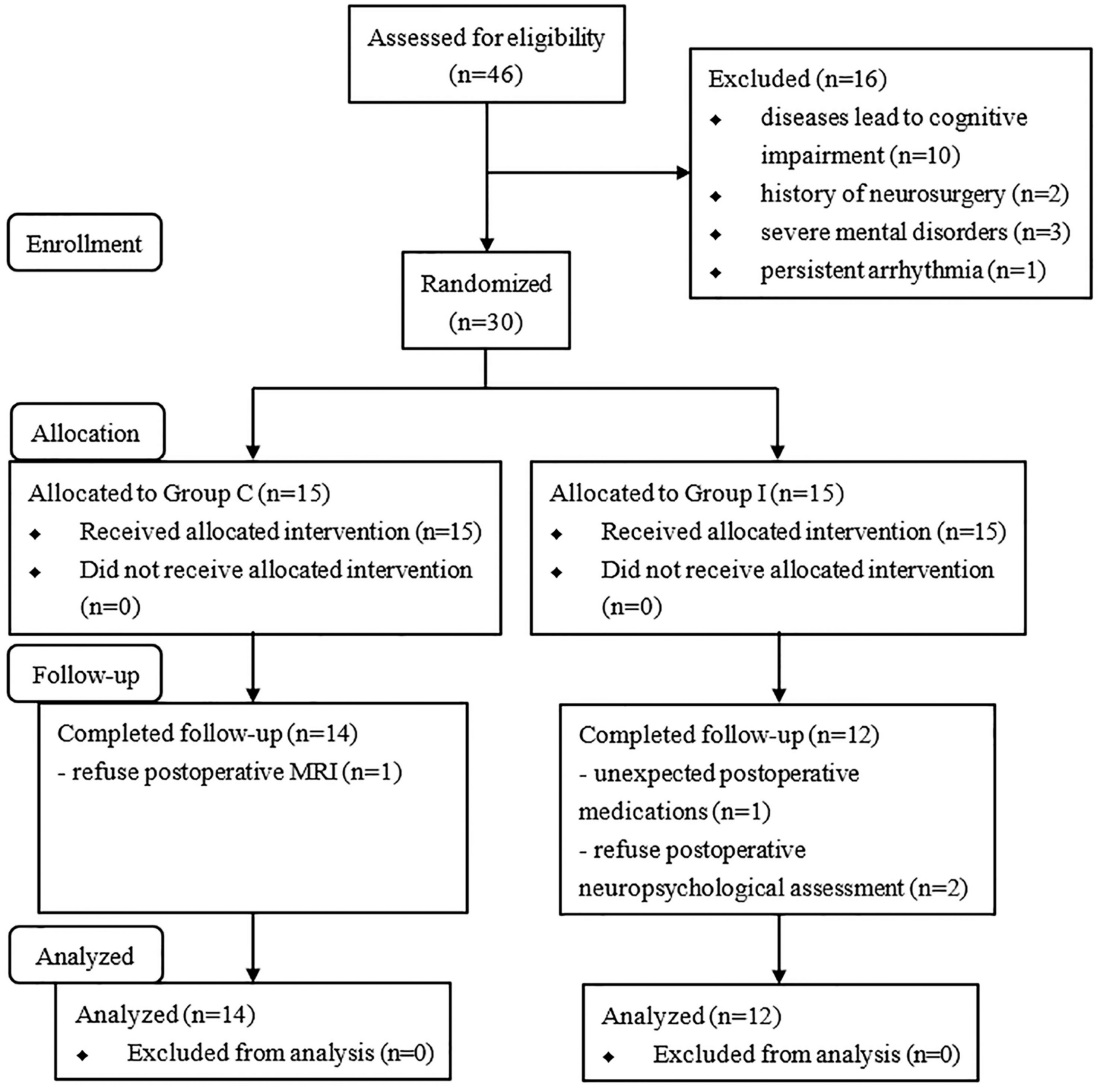
Flowchart of the study.

A total of 46 consecutive patients scheduled for spinal surgery were recruited, and 30 were ultimately included in the study and randomly assigned to the two groups (Figure 2). Four of the patients were excluded from the analysis (two for refusing the post-operative neuropsychological assessment, one for refusing to undergo MRI scanning, and one for unexpected post-operative medications). Therefore, 26 patients were included in the final analysis (14 in Group C and 12 in Group I). The characteristics of patients are presented in Table 1. No significant differences were observed in the demographic profiles of the two groups.

**Table 1:** The demographic characteristic data of patients.

**Characteristics**	**Group C (*n* = 14)**	**Group I (*n* = 12)**	**Effect size**	***p-*value**
Age (years)	70.84 ± 4.37	69.80 ± 7.15	0.18	0.70
Gender (male/female)	5/9	6/6	0.29	0.46
BMI (kg/m^2^)	25.54 ± 4.77	24.07 ± 1.79	0.41	0.38
ASA classification (I/II)	5/9	6/6	0.29	0.46
Education (years)	9.44 ± 3.05	10.05 ± 3.72	0.18	0.51
History of hypertension [*n* (%)]	4 (28.57)	3 (25.00)	0.08	0.84
History of diabetes [*n* (%)]	1 (7.14)	2 (16.67)	0.42	0.45
History of CAD [*n* (%)]	2 (14.29)	1 (8.33)	0.29	0.64
operative segment (segments)	2.00 (2.00~2.50)	2.00 (1.75~2.25)	0.30	0.43

*Data are mean ± SD, median (IQR), or frequencies*.

*BMI, body mass index; ASA, american society of anesthesiologists physical status; CAD, coronary artery disease; IQR, interquartile range*.

### Neuropsychological Assessment and Incidence of PND

The MoCA scores at T_0_ [1 day before surgery (baseline)] and T_2_ (7 days after surgery) and incidence of PND are presented in Table 2. The mean MoCA score was higher in Group I than in Group C. Although the incidence of PND determined based on *Z*-score showed no significant difference between the two groups, a trend toward significance was observed in Group I.

**Table 2:** Neuropsychological assessment and incidence of PND.

	**Group C (*n* = 14)**	**Group I (*n* = 12)**	**Effect size**	***p-*value**
MoCA score (T_0_)	21.78 ± 2.73	23.50 ± 2.51	0.66	0.17
MoCA score (T_2_)	22.56 ± 2.24	24.80 ± 2.09	1.03	0.04
PND [*n* (%)]	4 (28.57)	2 (16.67)	0.33	0.47

*Data are mean ± SD or frequencies*.

*PND, post-operative neurocognitive disorder; MoCA, Montreal Cognitive Assessment; T_0_, 1 day before surgery; T_2_, 7 days after surgery*.

*p < 0.05 was considered as statistically significant*.

### Anesthesia Management Based on Multimodal Brain Monitoring Alters ALFF

**Figure 3:**
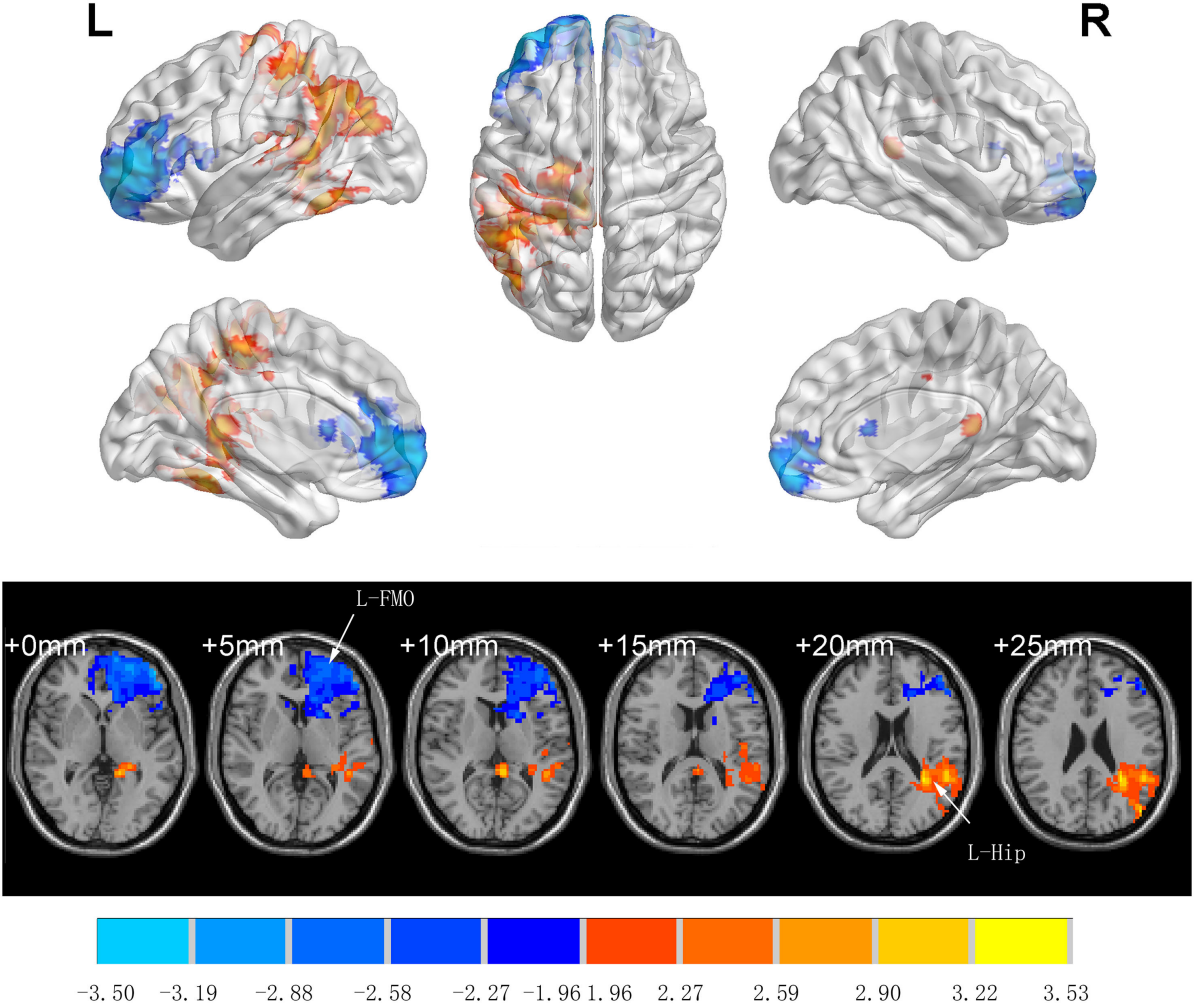
Significant differences in ALFF between Groups I and C 7 days after surgery (voxel *p* < 0.05, GRF-corrected). Increased ALFF was observed in the left middle temporal gyrus, left precentral gyrus, left paracentral lobule, left hippocampus, left parahippocampal gyrus and left precuneus, decreased ALFF was observed in the left orbital middle frontal gyrus, left orbital superior frontal gyrus, and left medial superior frontal gyrus between Group I and Group C. ALFF, amplitude of low-frequency fluctuation; GRF, gaussian random field.

There was no significant difference between groups in preoperative ALFF (GRF-corrected). Compared to Group C, patients in Group I had significantly higher ALFF values in the left middle temporal gyrus (TG), left precentral gyrus (L-PCG), left paracentral lobule, left hippocampus (L-Hip), left parahippocampal gyrus, and left precuneus 7 days after spinal surgery; meanwhile, ALFF was decreased in the left orbital middle FG, left orbital superior (S)FG, and left medial SFG (GRF-corrected; Table 3 and Figure 3).

**Table 3:** Regions showing significant difference in ALFF analysis between Group C and Group I at 7 days after surgery.

**Cluster name**	**Brain region**	**Cluster size**	**MNI coordinates**			**Effect size**	***t*-value**
			***x***	***y***	***z***		
Cluster 1	Left middle temporal gyrus	90	−51	−48	21	1.02	3.40
	Left precentral gyrus	72	−21	−27	54	0.69	3.35
	Left paracentral lobule	50	−12	−33	51	2.56	4.38
	Left hippocampus	28	−24	−36	1	0.91	3.09
	Left parahippocampal gyrus	26	−24	−36	−9	0.76	3.00
	Left precuneus	19	−15	−42	1	0.45	3.40
Cluster 2	Left orbital middle frontal gyrus	182	−36	60	−6	0.79	−3.77
	Left orbital superior frontal gyrus	132	−27	54	−3	0.74	−3.55
	Left medial superior frontal gyrus	98	−12	45	3	0.60	−3.09

*Data are mean ± SD or frequencies*.

ALFF, amplitude of low-frequency fluctuation; PND, post-operative neurocognitive disorder; MoCA, montreal cognitive assessment; T_0_, 1 day before surgery; T_2_, 7 days after surgery.

*p < 0.05 was considered as statistically significant*.

### Anesthesia Management Based on Multimodal Brain Monitoring Improves FC in the Aging Brain

**Figure 4:**
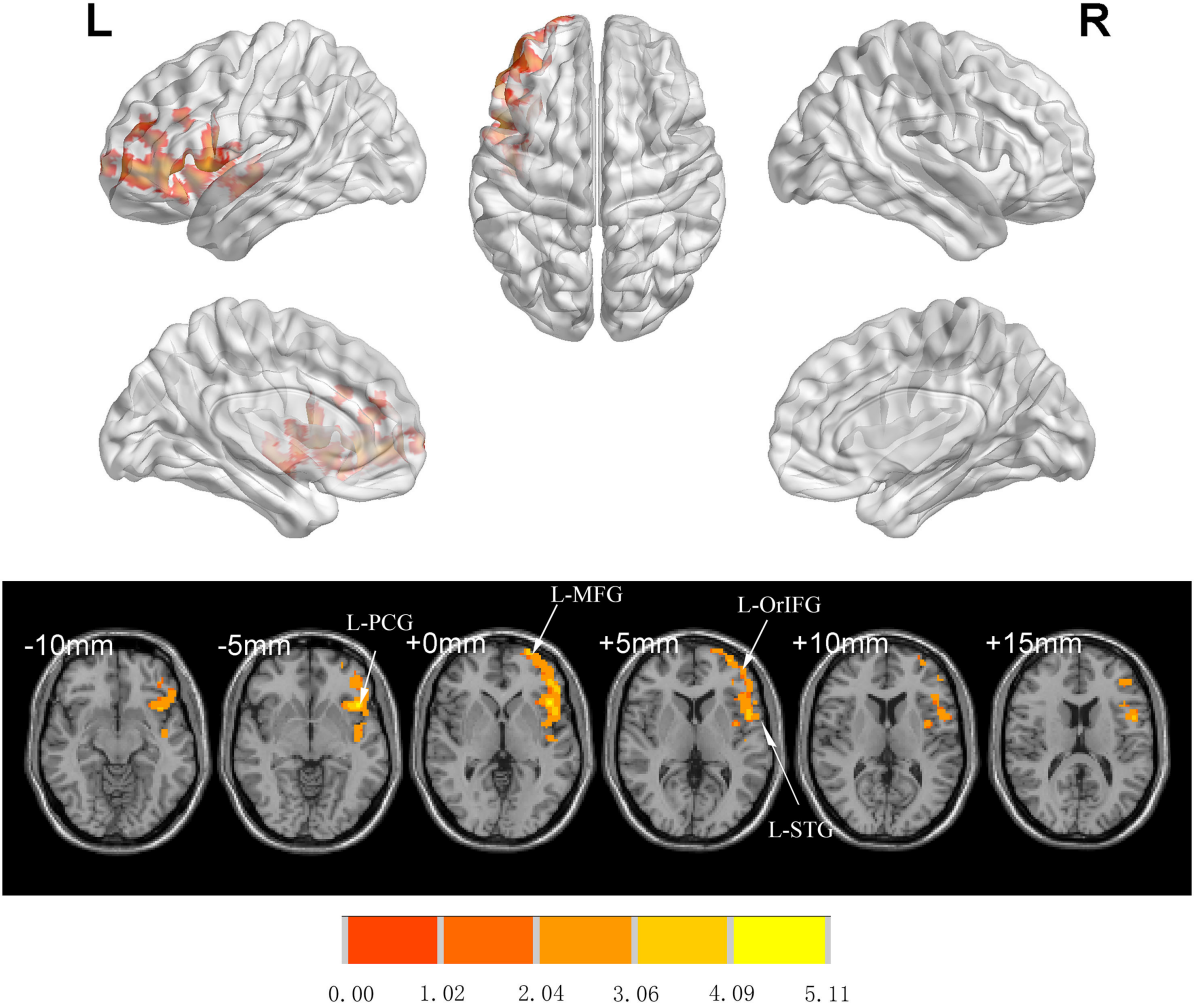
Significant differences in FC between Groups I and C 7 days after surgery (voxel *p* < 0.05, GRF-corrected). Increased FC was observed in the L-orIFG, L-MFG, L-STG, and L-PCG in Group I compared to Group C. L-PCG, left precentral gyrus; L-MFG, left middle frontal gyrus; L-OrIFG, left orbital inferior frontal gyrus; L-STG, left superior temporal gyrus; FC, functional connectivity; GRF, gaussian random field.

There was no significant difference between groups in preoperative FC (GRF-corrected). L-Hip was selected as the seed region for seed-based FC analysis. Compared to Group C, patients in Group I showed significantly increased FC in left orbital inferior FG (L-orIFG), left middle FG (L-MFG), left superior TG (L-STG), and L-PCG (GRF-corrected; Table 4 and Figure 4).

**Table 4:** Regions showing significant difference in FC analysis between Group C and Group I at 7 days after surgery.

**Cluster name**	**Brain region**	**Cluster size**	**MNI coordinates**			**Effect size**	***t*-value**
			***x***	***y***	***z***		
Cluster 1	Left orbital inferior frontal gyrus	144	−45	21	−3	0.99	8.64
	Left middle frontal gyrus	112	−45	45	1	0.89	3.66
	Left superior temporal gyrus	43	−45	−6	−6	0.43	3.38
	Left precentral gyrus	12	−48	6	21	0.31	2.60

*The results were considered significant at voxel p < 0.05 (two-tailed, GRF-corrected). A positive t-value represented an increase in FC when compared Group I to group C*.

*FC, functional connectivity; GRF, gaussian random field*.

### Markers of Inflammation Are Elevated After Surgery

There were no significant differences in pre- and post-operative CRP and LPS levels between Groups I and C (Table 5), and ΔCRP and ΔLPS (calculated as ΔCRP = CRP [T_1_] – CRP [T_0_] and ΔLPS = LPS [T_1_] –LPS [T_0_]) also did not differ between groups. However, compared to the baseline level (T_0_), CRP (*p* = 0.02) and LPS (*p* = 0.01) were significantly higher after surgery in Group C whereas in Group I, a significant increase was observed only for CRP (*p* = 0.01) with no significant change in LPS level 24 h after surgery.

**Table 5:** Serum inflammatory markers in two groups.

	**Group C (*n* = 14)**	**Group I (*n* = 12)**	**Effect size**	***p-*value**
**CRP (ng/ml)**				
CRP (T_0_)	1,627.76 ± 1153.46	4,104.58 ± 6955.50	0.50	0.33
CRP (T_1_)	18,369.97 ± 17691.66^*^	13,338.78 ± 8750.81^*^	0.36	0.44
ΔCRP(T_1_-T_0_)	16,742.21 ± 17179.62	9,324.20 ± 9,866.28	0.53	0.26
**LPS (ng/ml)**				
LPS (T_0_)	15.74 ± 15.96	19.16 ± 15.90	0.21	0.65
LPS (T_1_)	21.85 ± 14.51^*^	26.10 ± 17.87	0.26	0.58
ΔLPS (T_1_-T_0_)	6.11 ± 6.05	6.93 ± 12.88	0.08	0.86

*The serum CRP and LPS level were presented as mean ± SD*.

*CRP, C-reactive protein; LPS, lipopolysaccharide; T_0_, 1 day before surgery; T_1_, 24 h after surgery*.

* *Data were tested by paired sample t-test when compared T_1_ to T_0_ in same group. p < 0.05 was considered as statistically significant*.

### Functional Connectivity Is Negatively Correlated With Inflammation

Pearson's correlation coefficient was calculated to assess the correlation between Z-transformed (z)FC and the extent of changes in inflammatory markers (ΔCRP and ΔLPS) after transforming *t*-values with statistical significance to Z scores using Fisher's transformation. A negative correlation was observed between zFC and ΔCRP (*R* = −0.58, *p* = 0.01), whereas no significant correlation was observed between FC and ΔLPS (Table 6).

**Table 6:** Correlation between FC and inflammatory markers.

	***R*-value**	***p-*value**
ΔCRP	−0.58	0.01
ΔLPS	−0.03	0.92

*Data were analyzed by the Pearson correlation between zFC and Δ inflammatory markers. ΔCRP = CRP (T_1_) – CRP (T_0_), ΔLPS = LPS (T_1_) – LPS (T_0_). The zFC was calculated by the Fisher's Z transformation*.

*CRP, C-reactive protein; LPS, lipopolysaccharide; T_0_, 1 day before surgery; T_1_, 24 h after surgery*.

*p < 0.05 was considered as statistically significant*.

### Secondary Outcomes

Secondary outcomes, including fluid intake and output, intraoperative use of remifentanil, propofol and norepinephrine, NRS at 24 h after surgery, amount of oxycodone consumed, time of the first post-operative exhaust, and length of hospital stay, were compared between groups (Table 7). There were no significant differences in surgery duration, anesthesia duration, intraoperative fluid intake and output, use of anesthetics (including propofol and remifentanil), norepinephrine consumption, NRS, oxycodone consumption, and time of the first post-operative exhaust. The mean length of hospital stay was over 3 days shorter in Group I compared to Group C, although the difference was non-significant.

**Table 7:** Secondary outcomes.

	**Group C (*n* = 14)**	**Group I (*n* = 12)**	**Effect size**	***p-*value**
Surgery duration (min)	157.78 ± 65.33	154.00 ± 54.84	0.06	0.89
Anesthesia duration (min)	213.56 ± 67.44	222.90 ± 71.08	0.13	0.77
Fluid intake (ml)	1,555.56 ± 502.77	1,540.00 ± 313.40	0.04	0.93
Fluid output (ml)	879.44 ± 518.19	894.50 ± 476.25	0.03	0.95
Propofol (mg)	746.44 ± 297.00	703.00 ± 277.97	0.15	0.75
Remifentanil (mg)	3.25 ± 1.45	3.29 ± 1.10	0.03	0.94
Norepinephrine (mg)	0.49 ± 0.37	0.53 ± 0.33	0.11	0.79
NRS (T_1_)	2.00 (1.00~3.00)	1.5 (0.00~2.25)	0.42	0.35
Oxycodone consumption (mg)	2.00 (1.00~6.50)	1.00 (0.00~3.00)	0.73	0.13
PONV [*n* (%)]	3 (21.43)	2 (16.67)	0.15	0.76
First time for post-operative exhaust (h)	10.44 ± 3.75	11.30 ± 4.92	0.20	0.68
Hospital stays (days)	11.44 ± 2.92	8.70 ± 2.79	0.96	0.05

*All the data listed except for the NRS, oxycodone consumption and rate of PONV were normally distributed and presented as mean ± SD. NRS and oxycodone consumption data are presented as median (IQR). PONV data are presented as frequencies (%)*.

*IQR, interquartile range; NRS, numeric rating scale; PONV, post-operative nausea and vomiting*.

*p < 0.05 was considered as statistically significant*.

There were three cases of post-operative nausea and vomiting (PONV) in Group C and two cases in Group I, with no significant differences between the two groups (*p* = 0.76). No other adverse effects of surgery were observed. There were two patients in Group C with post-operative complications during hospitalization (CSF leakage and lower limb pain) but no complications in Group I.

## Discussion

In the present study, we demonstrated that anesthesia management based on multimodal brain monitoring in elderly patients undergoing spinal surgery significantly enhanced post-operative neuropsychological assessment scores, improved brain activity, and reduced the effects of surgery and anesthesia on FC in the cognition-related brain regions compared to the routine anesthesia management. The differences between groups may be attributable to changes in systemic levels of inflammatory markers caused by the surgery and anesthesia.

The present study focused on elderly patients undergoing spinal surgery. Compared to adolescent patients, elderly patients have an increased risk of cognitive dysfunction after the surgery, which increases with aging (Evered et al., 2018; Daiello et al., 2019). We previously reported that elderly patients undergoing spinal surgery had a significantly higher rate of cognitive decline without intraoperative anti-inflammatory management (e.g., ulinastatin; Zhang et al., 2018), suggesting that these patients were at risk for developing PND. However, the functional changes that occur in the brain in PND are unknown.

Post-operative cognitive function is influenced by several factors (Chernov et al., 2006; Slater et al., 2009; Hou et al., 2018). Oversedation is a risk factor for PND: patients over the age of 60 years undergoing total knee arthroplasty had lower MoCA scores when the BIS value was controlled at 40–50 as compared to 55–65 (Hou et al., 2018). However, as an EEG-based monitoring method, BIS value is not influenced solely by the sedation depth; for example, it may also be affected by pain stress. Inadequate analgesia can lead to β arousal (Kortelainen et al., 2012). Cerebral hypoperfusion and lower rSO_2_ are also associated with cognitive deficits. In a study of elderly patients undergoing coronal artery bypass graft surgery (Chernov et al., 2006), a significant correlation was observed between cognitive function and change in cerebral perfusion in the short term and at 6 months post-operation; and another study found that regional brain anoxia and long-term hypoxia were risk factors for post-operative cognitive decline (Slater et al., 2009). We, therefore, developed our management strategy based on the monitoring of these parameters. Our results showed that management based on multimodal brain monitoring improved the post-operative cognitive function, as evidenced by the increased MoCA score. The MoCA can detect mild cognitive changes with high sensitivity and specificity (Luis et al., 2009) and was therefore used to evaluate cognitive function. PND was diagnosed based on the *Z* score recommended by the International Study of Post-operative Cognitive Dysfunction (Moller et al., 1998). Although there was no significant difference in the rates of PND in the two groups, there was a meaningful tendency in Group I patients.

Neuroinflammation induced by anesthesia and surgery contributes to PND (Safavynia and Goldstein, 2018). Preclinical studies in mice have demonstrated that nuclear factor (NF)-κB signaling is activated after surgery and stimulates the release of proinflammatory cytokines that increase the permeability of the blood–brain barrier (Wang et al., 2011). An elevated CRP level has been observed in patients over the age of 60 after lumbar surgery (Repo et al., 2019). The combination of surgical trauma and enhanced cytokine release has been proposed as a major cause of neuroinflammation and PND (Dantzer, 2001; Kapila et al., 2014). The levels of inflammatory cytokines were shown to be increased in the central nervous system and peripheral blood following surgery (Hirsch et al., 2016). In our study, the increased serum levels of CRP after surgery reflected systemic inflammation responses, although LPS was increased only in the control group (Group C), indicating that the inflammatory response was diminished by the multimodal brain monitoring intervention, which may have contributed to the higher post-operative MoCA score in Group I.

We used rs-fMRI to evaluate brain network connectivity. Changes in ALFF reflect altered brain activity. Although the diagnostic value of ALFF has been demonstrated for AD, subjective cognitive decline, and amnestic MCI (Yang et al., 2018; Zhuang et al., 2020), few studies have examined ALFF in relation to the development of PND. Decreasing ALFF values have been observed in the precuneus, anterior cingulate cortex, parahippocampal gyrus, and temporal lobe of patients with AD with disease progression (Liu et al., 2014; Sheng et al., 2017; Yang et al., 2018). We also observed changes in ALFF in some of these brain regions, although some of our findings diverged from those of other studies: we found that ALFF was decreased in the Hip of patients in Group C, whereas an increase in ALFF was reported in the left Hip of patients with AD (Liu et al., 2014; Yang et al., 2018). The difference may be attributable to the distinct pathogenic mechanisms of AD vs. PND. The increased ALFF in the Hip of patients with AD may be a compensatory response in long-term neurodegenerative diseases (Yang et al., 2018). In contrast, PND is reversible, and the duration may be too short for long-term compensatory mechanisms to be activated. This may explain our observation that a decreased MoCA score was associated with a lower ALFF value in the Hip, which is the opposite of what has been demonstrated in patients with AD.

Intrinsic FC is more stable in the resting state than during task-activated synchronization (Cao et al., 2014). Intrinsic functional networks appear similar on a global level when compared with task-evoked networks (Cole et al., 2014), highlighting the importance of resting-state brain networks in cognitive function (Buckner et al., 2009). Several studies have suggested a link between functional brain networks and human cognition (Buckner et al., 2008; Song et al., 2008); the level of FC may have predictive value for cognitive performance. In patients with AD, reduced connectivity in brain networks was shown to be correlated with disease severity, and the connectivity was positively associated with executive function and language scores (Agosta et al., 2012), suggesting that FC patterns are related to cognitive performance.

When L-Hip was used as a seed region, the FC between L-Hip and L-orIFG, L-MFG, L-STG, and L-PCG was increased in patients who were managed based on multimodal brain monitoring; this was accompanied by an increase in MoCA score. The orbitofrontal cortex (OFC) is thought to play a key role in executive and cognitive functions (Kawamura et al., 2011). Post-mortem studies have revealed anatomic connections between the OFC and thalamus, anterior cingulate cortex, temporal lobe, and occipital lobe (Burks et al., 2018); and the MFG was found to be related to memory reclamation (Brincat and Miller, 2015). The human STG is part of the temporal lobe, which contributes to language function. A task-based fMRI study revealed that FC in the temporal lobe and other brain regions increased along with naming ability with the surgical effect in patients with temporal lobe epilepsy (Trimmel et al., 2018). However, language ability cannot be solely treated as an independent ability but correlated with cognitive function. In patients with multiple sclerosis, the thickness of the STG was shown to be correlated with cognitive decline (Achiron et al., 2013). The PCG, which functions as the motor center, is located between the central and precentral sulci. However, patients with MCI who received nutritional supplementation showed a stronger BOLD signal and higher MoCA score, suggesting that the PCG may also participate in cognitive regulation (Boespflug et al., 2018), which is supported by our findings. Higher FC values indicated stronger associations between the above-mentioned brain regions and L-Hip, which could explain the higher MoCA scores in patients who were managed based on multimodal brain monitoring.

In our previous study, we demonstrated that a single dose of LPS (2 mg/kg by intraperitoneal injection) induced long-term neuroinflammation (for up to 30 days) in the Hip of aged rats with upregulation of tumor necrosis factor (TNF)-α, interleukin (IL)-1β, and NF-κB mRNA expression (Fu et al., 2014). Thus, peripheral inflammation can cause long-term cognitive changes in aging rats. Meanwhile, the application of NF-κB pathway blocker pyrrolidine dithiocarbamic acid reduced the levels of inflammatory cytokines in the Hip and the expression of post-synaptic density (PSD)-95, and improved long-term cognitive function in aged rats exposed to LPS (Kan et al., 2016). FC in aged rats is related to cognitive function, which may be exacerbated by neuroinflammation induced by a systemic inflammatory response (Liu et al., 2021). In our study, a significant correlation was observed between FC and ΔCRP, and management based on multimodal brain monitoring may have prevented an increase in the post-operative LPS level. The results of the current study and our previous work indicate that management based on multimodal brain monitoring during surgery may improve brain FC by reducing neuroinflammation in aged patients (Fu et al., 2014; Kan et al., 2016; Liu et al., 2021).

There were several limitations to this study. First, we did not investigate the detailed mechanisms underlying the observed effects of the anesthesia management strategy because of ethical concerns. Second, all of the patients enrolled in this study underwent spinal surgery; whether our results are generalizable to other types of surgery remains to be determined. Finally, the sample size was small, which could explain the lack of differences in some secondary outcome measures.

In conclusion, the present study demonstrated that anesthesia management based on multimodal brain monitoring under general anesthesia may improve the post-operative cognitive function and brain function connectivity in elderly patients undergoing spinal surgery compared to routine anesthesia management, as evidenced by increased brain activity (ALFF), enhanced FC, higher MoCA score, and reduced systemic inflammation. The extent of post-operative systemic inflammation was negatively associated with the FC enhancement and may be accompanied by a lower MoCA score. Our findings provide a basis for more effective management of elderly patients who undergo surgery to reduce the risk of cognitive disorders and improve brain function.

## Data Availability Statement

The raw data supporting the conclusions of this article will be made available by the authors, without undue reservation.

## Ethics Statement

The studies involving human participants were reviewed and approved by Institutional Review Board of Xuanwu Hospital, Capital Medical University. The patients/participants provided their written informed consent to participate in this study.

## Author Contributions

SY, WX, JL, and TW: study design. SY, WX, HW, YL, and SF: study performance. SY, WX, YL, and JL: data analysis. SY, WX, YL, and TW: manuscript writing. TW and WX: manuscript revision. All authors contributed to the article and approved the submitted version.

## Conflict of Interest

The authors declare that the research was conducted in the absence of any commercial or financial relationships that could be construed as a potential conflict of interest.
